# *Plasmodium falciparum* Parasites Are Killed by a Transition State Analogue of Purine Nucleoside Phosphorylase in a Primate Animal Model

**DOI:** 10.1371/journal.pone.0026916

**Published:** 2011-11-11

**Authors:** María B. Cassera, Keith Z. Hazleton, Emilio F. Merino, Nicanor Obaldia, Meng-Chiao Ho, Andrew S. Murkin, Richard DePinto, Jemy A. Gutierrez, Steven C. Almo, Gary B. Evans, Yarlagadda S. Babu, Vern L. Schramm

**Affiliations:** 1 Department of Biochemistry, Albert Einstein College of Medicine, Yeshiva University, Bronx, New York, United State of America; 2 Tropical Medicine Research, Malaria Drug and Vaccine Evaluation Center, Gorgas Memorial Institute of Health Studies, Panama City, Panama; 3 Waters Corporation, Parsippany, New Jersey, United State of America; 4 Carbohydrate Chemistry Group, Industrial Research Ltd., Lower Hutt, New Zealand; 5 Department of Biological Sciences, BioCryst Pharmaceuticals Inc., Birmingham, Alabama, United State of America; 6 Department of Chemistry, University at Buffalo, Buffalo, New York, United State of America; Stanford University, United States of America

## Abstract

*Plasmodium falciparum* causes most of the one million annual deaths from malaria. Drug resistance is widespread and novel agents against new targets are needed to support combination-therapy approaches promoted by the World Health Organization. *Plasmodium* species are purine auxotrophs. Blocking purine nucleoside phosphorylase (PNP) kills cultured parasites by purine starvation. DADMe-Immucillin-G (BCX4945) is a transition state analogue of human and *Plasmodium* PNPs, binding with picomolar affinity. Here, we test BCX4945 in *Aotus* primates, an animal model for *Plasmodium falciparum* infections. Oral administration of BCX4945 for seven days results in parasite clearance and recrudescence in otherwise lethal infections of *P. falciparum* in *Aotus* monkeys. The molecular action of BCX4945 is demonstrated in crystal structures of human and *P. falciparum* PNPs. Metabolite analysis demonstrates that PNP blockade inhibits purine salvage and polyamine synthesis in the parasites. The efficacy, oral availability, chemical stability, unique mechanism of action and low toxicity of BCX4945 demonstrate potential for combination therapies with this novel antimalarial agent.

## Introduction

**Figure 1:**
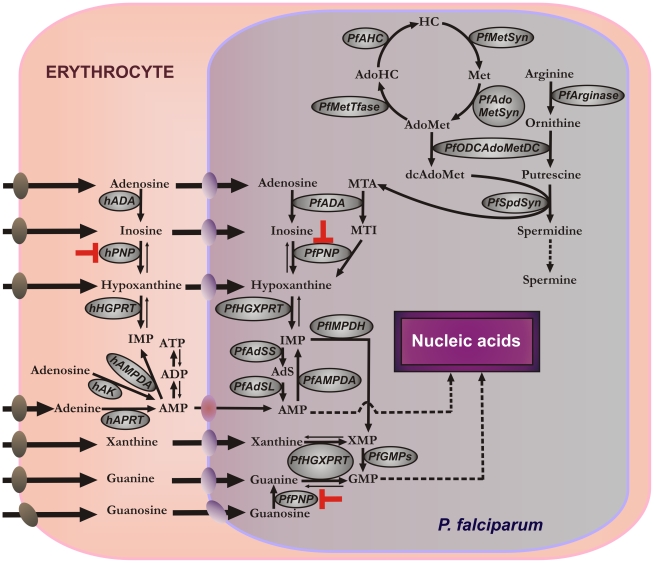
Purine and polyamine metabolisms in *P. falciparum*-infected human erythrocytes. **Purine pathway: AMP**, adenosine 5′-monophosphate; **ADP**, adenosine 5′-diphosphate; **ATP**, adenosine 5′-triphosphate; **IMP**, inosine 5′-monophosphate; **XMP**, xanthosine 5′-monophosphate; **GMP**, guanosine 5′-monophosphate; **MTA**, methylthioadenosine; **MTI**, methylthioinosine; **AdS**, adenylosuccinate; **hADA**, human adenosine deaminase; **hPNP**, human purine nucleoside phosphorylase; **hHGPRT**, human hypoxanthine-guanine phosphoribosyl transferase; **hAK**, human adenosine kinase; **hAMPDA**, human adenosine 5′-monophosphate deaminase; **hAPRT**, human adenine phosphoribosyl transferase; ***Pf*** **ADA**, *P. falciparum* adenosine deaminase; ***Pf*** **PNP**, *P. falciparum* purine nucleoside phosphorylase; ***Pf*** **HGXPRT**, *P. falciparum* hypoxanthine-guanine-xanthine phosphoribosyl transferase; ***Pf*** **AMPDA**, *P. falciparum* adenosine 5′-monophosphate deaminase; ***Pf*** **IMPDH**, *P. falciparum* inosine 5′-monophosphate dehydrogenase; ***Pf*** **GMPs**, *P. falciparum* guanosine 5′-monophosphate synthase; ***Pf*** **AdSS**, adenylosuccinate synthase; ***Pf*** **AdSL**, adenylosuccinate lyase. **Polyamine pathway: AdoMet**, *S*-adenosylmethionine; **AdoHC**, *S*-adenosylhomocysteine; **HC**, homocysteine; **Met**, methionine; **dcAdoMet**, decarboxylated *S*-adenosylmethionine; ***Pf*** **SpdSyn**, *P. falciparum* spermidine synthase; ***Pf*** **ODCAdoMetDC**, *P. falciparum* ornithine decarboxylase*/S-*adenosylmethionine decarboxylase; ***Pf*** **MetTfase**, *P. falciparum* methyltransferase(s); ***Pf*** **AHC**, *P. falciparum S*-adenosyl homocysteinase; ***Pf*** **MetSyn**, *P. falciparum* methionine synthase; ***Pf*** **AdoMetSyn**, *P. falciparum S*-adenosylmethionine synthase. The metabolically favored direction is indicated with bold arrows on reversible steps. The metabolic step inhibited by BCX4945 is indicated in red. Nucleoside/nucleobase transporters are indicated on each membrane: human erythrocyte nucleoside transporter (brown), *P. falciparum* NT1 transporter (blue) and yet-to-be characterized adenosine 5′-monophosphate transporter (purple).

*Plasmodium* parasites are purine auxotrophs and require preformed purine bases for synthesis of nucleotides, cofactors, and nucleic acids [1]. Purine salvage in *P. falciparum* uses hypoxanthine formed in erythrocytes or in parasites by the sequential actions of adenosine deaminase (hADA, *Pf*ADA) and/or purine nucleoside phosphorylase (hPNP, *Pf*PNP) [2] (Figure 1). Hypoxanthine, inosine and adenosine are transported from erythrocytes into parasites by the equilibrative nucleoside transporter (*Pf*NT1) [3], [4]. In erythrocytes, hypoxanthine and ATP are in dynamic metabolic exchange via ADP, AMP, IMP, inosine and adenosine. Hypoxanthine in the parasite is converted to IMP by hypoxanthine-guanine-xanthine phosphoribosyl-transferase (*Pf*HGXPRT) to provide inosine monophosphate (IMP), a precursor for all required purines. Host and parasite PNPs are essential for the formation of hypoxanthine, making PNPs a target for the purine salvage pathway.

The polyamine biosynthetic pathway in *P. falciparum* depends on *Pf*ADA and *Pf*PNP to recycle 5′-methylthioadenosine (MTA), a product of polyamine synthesis (Figure 1). Its removal is necessary for the cell to perform polyamine metabolism, since MTA is a strong inhibitor of spermine synthase, spermidine synthase and of ornithine decarboxylase [5], [6], [7]. Primates encode a specific MTA phosphorylase for this purpose, but this activity is not found in *P. falciparum*. Instead, the *Pf*ADA deaminates MTA to 5′-methylthioinosine (MTI), a metabolite not found in mammals [2] (Figure 1). *Pf*PNP converts MTI to hypoxanthine for subsequent conversion to IMP. Blocking *Pf*PNP therefore has the potential to block purine salvage by preventing hypoxanthine formation and to disrupt MTA recycling in the parasite, and thereby interfere with polyamine metabolism. Polyamines neutralize charge on nucleic acids and are essential to rapidly proliferating cells like *P. falciparum*, where they are a major metabolite [8]. Polyamine synthetic pathways are important for parasites since mammalian erythrocytes do not synthesize polyamines and only trace amounts are found in serum [9].

Purine salvage in *P. falciparum* relies on hypoxanthine salvage and can be disrupted with transition-state analogue inhibitors effective against both human and *Plasmodium* PNPs (Figure 1). Thus, inhibitors of human and *Plasmodium* PNPs are lethal for *P. falciparum* cultured *in vitro* [10], [11]. Although PNP is present both in parasites and human erythrocytes, genetic evidence suggests that *Pf*PNP is critical for *in vitro* growth. *P. falciparum* genetically disrupted in PNP have increased purine requirements and are unable to thrive *in vitro* at physiological concentrations of hypoxanthine [12]. *P. yoelii* parasites (a rodent-specific species) genetically disrupted in PNP are attenuated when infecting mice [13].

*P. falciparum* is the most lethal of malaria parasites in humans and it has narrow host specificity. *Aotus* monkeys provide a non-human primate model for testing the efficacy of PNP transition-state analogues against this parasite. Here, we report that inhibition of PNP by orally administrated 4′-deaza-1′-aza-2′-deoxy-1′-(9-methylene)-Immucillin-G (here named BCX4945), clears the blood of *P. falciparum* in *Aotus* monkeys followed by recrudescence when treatment is stopped. BCX4945 causes depletion of hypoxanthine from *Aotus* blood, demonstrating inhibition of both hPNP and *Pf*PNP *in vivo* [11]. Moreover, we apply metabolic tests to establish that PNP inhibition by BCX4945 blocks purine salvage, and demonstrate that polyamine synthesis is also reduced in the human malaria parasite. We determine the molecular mechanism of BCX4945 inhibition by providing the crystal structures of BCX4945 with human and *P. falciparum* PNPs. This is the first time that this class of compounds is proved to be effective in a primate *in vivo* malaria model.

## Results

### PNP as an anti-malarial target

**Figure 2:**
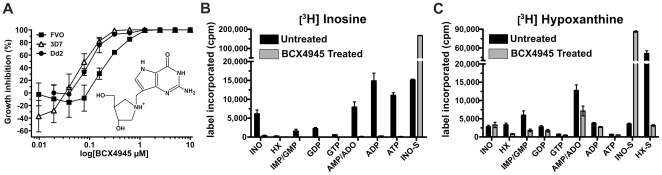
BCX4945 inhibits PNP to block inosine salvage. (**A**) Chemical structure and effect of BCX4945 on *in vitro* growth of different *P. falciparum* strains. Parasites were incubated in the presence of the indicated concentrations of BCX4945 for 72 h at 1% hematocrit, followed by DNA quantitation. IC_50_ values were calculated from fits (Origin software) to the overall response curve. The graph is constructed by individual point connections. (**B, C**) Counts per minute (cpm) levels of [^3^H]inosine and [^3^H]hypoxanthine metabolically incorporated into purine derivatives. *P. falciparum* infected-red blood cells in schizont and trophozoite stages were metabolically labeled with [^3^H]inosine or [^3^H]hypoxanthine in the absence or presence of 10 µM of BCX4945. Labeled inosine (INO-S) and hypoxanthine (HX-S) present in the supernatant. Means ± s.d. from triplicates are represented.

Purine salvage pathways have been targets for anti-malarials since the discovery that *Plasmodium* parasites are purine auxotrophs [14]. Adequate inhibition of PNP requires inhibitors with extraordinary affinity as the enzyme is present at high levels in the host erythrocytes and in *P. falciparum* [15]. Thus, in *P. falciparum in vitro* cultures, it is reported that the half-maximum inhibitory concentration (IC_50_) for PNP inhibitors increases with the hematocrit [11]. Immucillins are powerful picomolar transition-state analogue inhibitors of both human and *Plasmodium* PNPs [16]. They are orally available and of low toxicity to animals and humans [17]. We selected BCX4945 as an inhibitor for *in vivo* trials because of its high affinity for both host and parasite PNPs (7 pM and 890 pM *K*_d_ values, respectively) [18]. We assayed BCX4945 with different *P. falciparum* strains at human physiological concentration of hypoxanthine (<10 µM) [19] to determine the IC_50_ (Figure 2A). Parasitaemia was assessed by measuring PicoGreen fluorescence from nucleic acids of the parasites [20]. BCX4945 inhibited *P. falciparum* growth *in vitro* and the IC_50_ values were similar in a drug-sensitive strain 3D7 (164±20 nM) [10] and in a chloroquine/mefloquine-resistant strain Dd2 (130±22 nM) and a chloroquine/quinine resistant strain FVO (202±27 nM). These values were obtained at 1% hematocrit. At low concentrations BCX4945 caused increased parasitaemia relative to controls. Erythrocyte PNP is inhibited preferentially at low BCX4945 levels, resulting in increased cellular inosine to cause enhanced parasite growth [11].

Inhibition of both human and *P. falciparum* PNPs blocks inosine conversion into hypoxanthine and induces purine starvation, thus inhibiting parasite proliferation [2], [11]. Metabolic studies using [^3^H]inosine in the presence BCX4945 showed complete inhibition of the radiolabeled inosine incorporation into the purine pool in *P. falciparum*-infected erythrocytes (Figure 2B). Radiolabeled inosine accumulated in the culture media of treated parasites, since it is exclusively metabolized by human and *Pf*PNPs (Figure 2B, INO-S).

In contrast to the block in [^3^H]inosine incorporation in the presence BCX4945, [^3^H]hypoxanthine incorporation continues at reduced levels, except into inosine in the supernatant (Figure 2C, INO-S). Hypoxanthine, but not inosine, can bypass PNP inhibition (Figure S1). Supernatants analyzed from *P. falciparum*-infected erythrocytes labeled with [^3^H]hypoxanthine revealed that hypoxanthine is converted to [^3^H]IMP and to inosine by an IMP phosphohydrolase (Figure 2C). Thus, when PNP is blocked, added [^3^H]hypoxanthine accumulates as [^3^H]inosine in the supernatant. Erythrocytes have active equilibrative nucleoside transporters [21] and the efflux of labeled inosine reflects the inability of cells to convert inosine to hypoxanthine when PNP is inhibited.

Metabolite levels of adenosine, MTA, inosine, MTI and hypoxanthine were quantitated in *P. falciparum*-infected human erythrocytes and uninfected-erythrocytes, with and without BCX4945-treatment. The supernatants from each condition were also analyzed. We used liquid chromatography-mass spectrometry (LC-MS) with deuterated internal standards to measure absolute metabolite concentrations. No hypoxanthine was found in BCX4945-treated infected erythrocytes or the supernatant (Table 1). However, inosine accumulated to 60 µM in the cellular supernatant when PNPs were blocked (Table 1). In contrast, inosine was not detected in normal infected erythrocytes, showing the robust activity of PNPs (Table 1). MTI is a metabolite only of the parasite and is a substrate for *Pf*PNP. Hence, MTI concentration increased in the supernatant of BCX4945-treated infected human erythrocytes, also demonstrating inhibition of *Pf*PNP (Table 1).

**Table 1:** Polyamine and purine levels in cultured *P. falciparum* infected-erythrocytes and human erythrocytes after 24 h treatment with 5 µM BCX4945.

		Infected-erythrocytes (nmoles/mg protein)		Erythrocytes (nmoles/mg protein)	
	Metabolite	Control	BCX4945-treated	Control	BCX4945-treated
**Cell pellet**	**Putrescine**	0.70±0.05	0.2±0.1 (0.01)	0	0
	**Spermidine**	3.9±0.3	1.1±0.3 (0.02)	0.060±0.004	0.060±0.003
	**Spermine**	0.25±0.02	0.07±0.03 (0.01)	0.035±0.002	0.030±0.002
	**Adenosine**	12±6	10±4	5±2	2.60±0.03 (0.2)
	**Inosine**	24±6	19±5	0	37±6 (0.04)
	**Hypoxanthine**	313±121	0 (0.007)	0	0
	**MTA**	0.7±0.2	0.6±0.1	0	0
	**MTI**	0.7±0.3	0.6±0.1	0	0
	**BCX4945**	0	20±6 (0.004)	0	36±12 (0.07)

		Infected- erythrocytes (µM)		Erythrocytes (µM)	
	Metabolite	Control	BCX4945-treated	Control	BCX4945-treated
**Supernatant**	**Adenosine**	1.5±0.4	0.4±0.2 (0.08)	0.3±0.1	0.6±0.2 (0.2)
	**Inosine**	0	61±5 (0.0001)	0	8±3 (0.08)
	**Hypoxanthine**	21±7	0 (0.004)	0.72±0.1	0 (0.04)
	**MTA**	0	0	0	0
	**MTI**	0.10±0.04	0.3±0.1 (0.03)	0	0
	**BCX4945**	0	5±0.6 (0.0002)	0	4.0±0.2 (0.01)

Data are mean ± s.d. from two experiments, *p* values are in parentheses.

*P. falciparum* requires PNP to recycle MTI, a product of polyamine metabolism in parasites but not found in human metabolism. We explored the biochemical block by BCX4945 to determine if it perturbed polyamine metabolism in parasites (Figure 1). Putrescine, spermidine and spermine in infected-human erythrocytes were significantly reduced by BCX4945 treatment (Table 1). Thus, inhibition of *Pf*PNP also inhibits polyamine metabolism in the parasites. Polyamines are a major metabolite in malaria parasites, estimated to be ∼14% of the small molecule metabolites [8]. However, strategies targeted specifically to polyamines have not found success in antimalarial therapy, supporting purine metabolism as the primary target of BCX4945 [9], [22].

### Therapeutic efficacy of BCX4945 in *P. falciparum* infection *in vivo*

**Figure 3:**
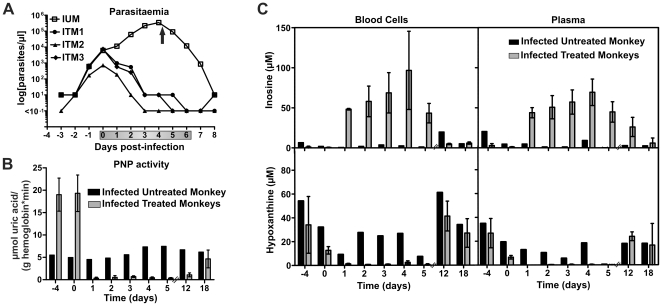
Oral administration of BCX4945 inhibits PNP and clears *P. falciparum* from infected *Aotus* monkeys. (**A**) Parasitaemia in infected untreated monkey (IUM, *n* = 1) or infected treated monkeys (ITM, *n* = 3). The arrow indicates mefloquine treatment to cure the infected untreated control monkey. Grey bar on the x-axis indicates days of treatment. (**B**) Blood cell PNP activity was assayed from untreated (*n* = 1) and treated monkeys (*n* = 3, means ± s.d.). Each sample was analyzed in triplicate. (**C**) Samples from blood cells and plasma were analyzed by UPLC-MS/MS. The (−4) time point indicates blood was taken before the monkey was infected. The (0) time point indicates that blood was drawn before the treatment started. Day 1 reflects parasitaemia or metabolite levels 24 h after the first dose and the metabolic effect of two BCX4945 doses within 24 h (also for days 2 to 5). Days 12 and 18 are counted from the start of treatment.

Encouraged by its potency in inhibiting *P. falciparum* proliferation *in vitro*, we tested BCX4945 for efficacy in *Aotus lemurinus lemurinus* monkeys infected with *P. falciparum* (FVO strain). In *Aotus* primates (Owl monkeys) infection by *P. falciparum* FVO is consistently lethal using the present protocol and without antimalarial intervention [23]. BCX4945 orally administered (50 mg kg^−1^) twice a day for seven days cleared *P. falciparum* infections between the fourth and the seventh day of treatment (Figure 3A). Monkeys remained parasite-negative for up to nine days post-treatment (Table 2). All three BCX4945-treated monkeys eventually recrudesced when treatment was terminated; however, a lower rate of parasitic growth was observed (Table 2). No signs of toxicity were observed during the study period (30 days after the first dose). Transient decreases in hemoglobin and hematocrit detected at day 12 post-infection, and increased glutamate pyruvate transaminase activity were attributed to the malaria infection (Table S1, tab PD-2).

**Table 2:** Parasitaemia values of infected-*Aotus* monkeys orally treated with 50 mg kg^−1^ of BCX4945 twice a day for 7 days.

	Day #	Control (IUM)	ITM 1	ITM 2	ITM 3
	**−1**	0.6	0.7	0.7	0.5
**Days of treatment**	**0**	6.2	7.2	0.7	7.5
	**1**	11.5	1	0.2	0.6
	**2**	62	0.6	0.01	0.3
	**3**	221	0.01	0	0.01
	**4**	350*	0.01	0	0.01
	**5**	91†	0.01	0	0
	**6**	12†	0	0	0
**Days Post-treatment**	**1**	0.9	0	0	0
	**2**	0.01	0.01	0	0
	**3**	0	0.7	0	0.01
	**4**	0	2.2	0	0.01
	**5**	0	46	0	0.3
	**6**	0	44*	0.01	0.2
	**7**	0	1.9	0.3	0.7
	**8**	0	0	0.2	1
	**9**	0	0	3	7.2
	**10**	cured	0	3.5	17.8
	**11**		cured	11.3	92*
	**12**			7.8	23.4
	**13**			10.8*	1.5

Parasites per µl×10^3^.

*One dose of mefloquine (40 mg kg^−1^);

† one dose of artesunic acid (33 mg kg^−1^). (IUM), Infected untreated monkey. (ITM), Infected treated monkey.

The action of BCX4945 caused inhibition of PNP and disruption of purine salvage in *P. falciparum*-infected *Aotus*. PNP activity was assayed in infected-blood cell pellets. Blood samples from an infected untreated monkey showed increased PNP activity as the parasitaemia increased (Figure 3B) while treated animals showed approximately 98% PNP inhibition during the treatment period (Figure 3B). Eleven days after the last dose of BCX4945, only ∼25% of the PNP enzymatic activity was regained (Figure 3B, day 18). Earlier studies in mice had demonstrated that picomolar PNP inhibitors remain bound to PNP for the life-time of the erythrocyte as a consequence of tight-binding to the enzyme. Thus, the kinetics of PNP activity return in blood is equal to the rate of hematopoiesis [24]. In *Aotus* infected with *P. falciparum*, the rate of erythrocyte replacement increases as a consequence of the anemia caused by the malaria infection (Table S1), as the normal erythrocytes lifespan in monkeys is 86 to 105 days [25]. Thus, PNP activity regain from BCX4945 treatment in *Aotus* blood was increased by *P. falciparum* infection, interpreted as a consequence of altered hematopoiesis rates (Figure S2).

### Purines in *P. falciparum-*infected *Aotus*

Exogenous hypoxanthine provides a metabolic by-pass to PNP inhibition (Figure 1 and S1). Thus, we characterized hypoxanthine levels in blood cells and plasma from *A. l. lemurinus* monkeys before and during *P. falciparum* infection (Table 3). In human volunteers, plasma hypoxanthine is reported to be 2.7 µM (*n* = 20) [26]. Serum hypoxanthine in *Aotus trivirgatus* monkeys is reported to be in the range of 31 to 192 µM [27]. Our findings are similar in *A. l. lemurinus* monkeys where hypoxanthine is the major purine base in plasma and blood cells (*n* = 14, plasma: 0 to 160 µM, mean = 40 µM; blood cells: 19 to 140 µM, mean = 64 µM; Table 3). After *Aotus* monkeys were infected with *P. falciparum* both plasma and cellular hypoxanthine significantly dropped (plasma: 0 to 40 µM, mean = 6 µM; blood cells: 0 to 83 µM, mean = 21 µM; Table 3).

**Table 3:** Hypoxanthine and inosine levels in *Aotus* monkeys' plasma and blood cells before and during *P. falciparum* infection*.

	Hypoxanthine (µM)				Inosine (µM)			
	Plasma		Blood cells		Plasma		Blood cells	
Monkey #	Before infection	During infection	Before infection	During infection	Before infection	During infection	Before infection	During infection
1	76.3	40.7	140.8	83.0	0.0	0.0	13.1	7.3
2	15.9	2.1	49.8	45.5	0.0	0.0	5.0	0.0
3	0.0	0.0	20.6	21.1	0.0	0.0	1.2	0.0
4	13.3	0.0	93.8	0.0	0.0	0.0	1.4	0.0
5	163.5	0.0	86.3	0.0	0.0	0.0	0.3	0.0
6	52.3	0.0	105.5	25.1	0.0	0.0	4.3	0.0
7	13.1	0.0	38.9	40.8	0.0	0.0	0.9	0.0
8	59.0	0.0	63.7	6.5	0.0	0.0	1.1	0.0
9	28.2	0.0	47.0	0.2	0.0	0.0	3.4	0.0
10	0.0	0.0	53.3	5.9	0.0	0.0	2.6	3.3
11	63.8	20.1	93.4	33.5	18.5	4.2	4.7	1.3
12	40.4	7.1	61.6	16.3	5.6	0.8	2.3	0.9
13	23.7	7.7	21.5	10.5	1.7	1.6	0.5	0.0
14	16.4	4.8	19.2	11.2	1.3	0.7	0.3	0.5

*Samples were collected before monkeys were infected with *P. falciparum* and four days after infection started.

Inosine is also abundant in normal *Aotus* and was found mainly in the blood cells (plasma: 0 to 18 µM, mean = 2 µM; blood cells: 0.3 to 13 µM, mean = 3 µM; Table 3). Similar to hypoxanthine, inosine levels dropped during *P. falciparum* infection (plasma: 0 to 4 µM, mean = 0.5 µM; blood cells: 0 to 7 µM, mean = 1 µM; Table 3). Treatment with BCX4945 to inhibit PNP activity caused inosine concentration to increase above 50 µM in *Aotus* plasma and blood cells while hypoxanthine concentration dropped below 0.3 µM (Figure 3C). Although BCX4945 treatment caused inosine accumulation, inosine cannot be converted to hypoxanthine in the presence of BCX4945. Adenosine is also a purine precursor for malaria parasites since it is converted to inosine by *Pf*ADA. Both plasma and blood cell adenosine levels were low (0.5 to 2 µM) and did not vary significantly with BCX4945 treatment. However, in untreated *Aotus*, adenosine concentration dropped as the infection progressed (Figure S3).

A human physiological substrate for PNP is 2′-deoxyguanosine and as expected, BCX4945 treatment increased the levels of 2′-deoxyguanosine both in plasma and in blood cells (Figure S3). Purines in *Aotus* blood returned to normal levels 18 days post-BCX4945 treatment even though PNP activity in erythrocytes remains 75% inhibited (Figure 3B, 3C, and Figure S3).

### BCX4945 pharmacokinetics and pharmacodynamics

Pharmacokinetics of BCX4945 were determined in uninfected and in infected *Aotus* treated orally (50 mg kg^−1^), or intravenously (10 mg kg^−1^) once a day for three days. Oral bioavailability of BCX4945 was 28% (*F*) and reached approximately 6 µM in plasma within 1 h of the first dose and up to 15 µM as the maximum concentration (*C*_max_) independent of infection (Figure S4). The *C*_max_ value for BCX4945 was achieved earlier in infected-monkeys (2 h versus 6 h in uninfected-monkeys). At 24 h following the first dose, BCX4945 was found in plasma at ∼1.2 µM in orally treated uninfected-animals. However, the half-life (t ½) in plasma was similar in animals orally (1.2 h) and intravenously (1.3 h) treated and these values were not affected by *P. falciparum* infection. Infected *Aotus* treated with once a day BCX4945 oral dosing (50 mg kg^−1^) for three days showed a 99% reduction of parasitaemia that returned rapidly following completion of treatment, as BCX4945 is cleared in 8 h from plasma (Figure S4 and S5A). Metabolite profiles in these monkeys showed that hypoxanthine decreased to near zero when BCX4945 is present (Figure S5B). Hypoxanthine remained near zero in plasma (0.3±0.2 µM) and BCX4945 persisted (0.6±0.2 µM) throughout the treatment period when BCX4945 was given twice a day at 50 mg kg^−1^ per dose, explaining the enhanced antimalarial effect (Figure S3).

### Structural basis for BCX4945 inhibition of PNPs

**Figure 4:**
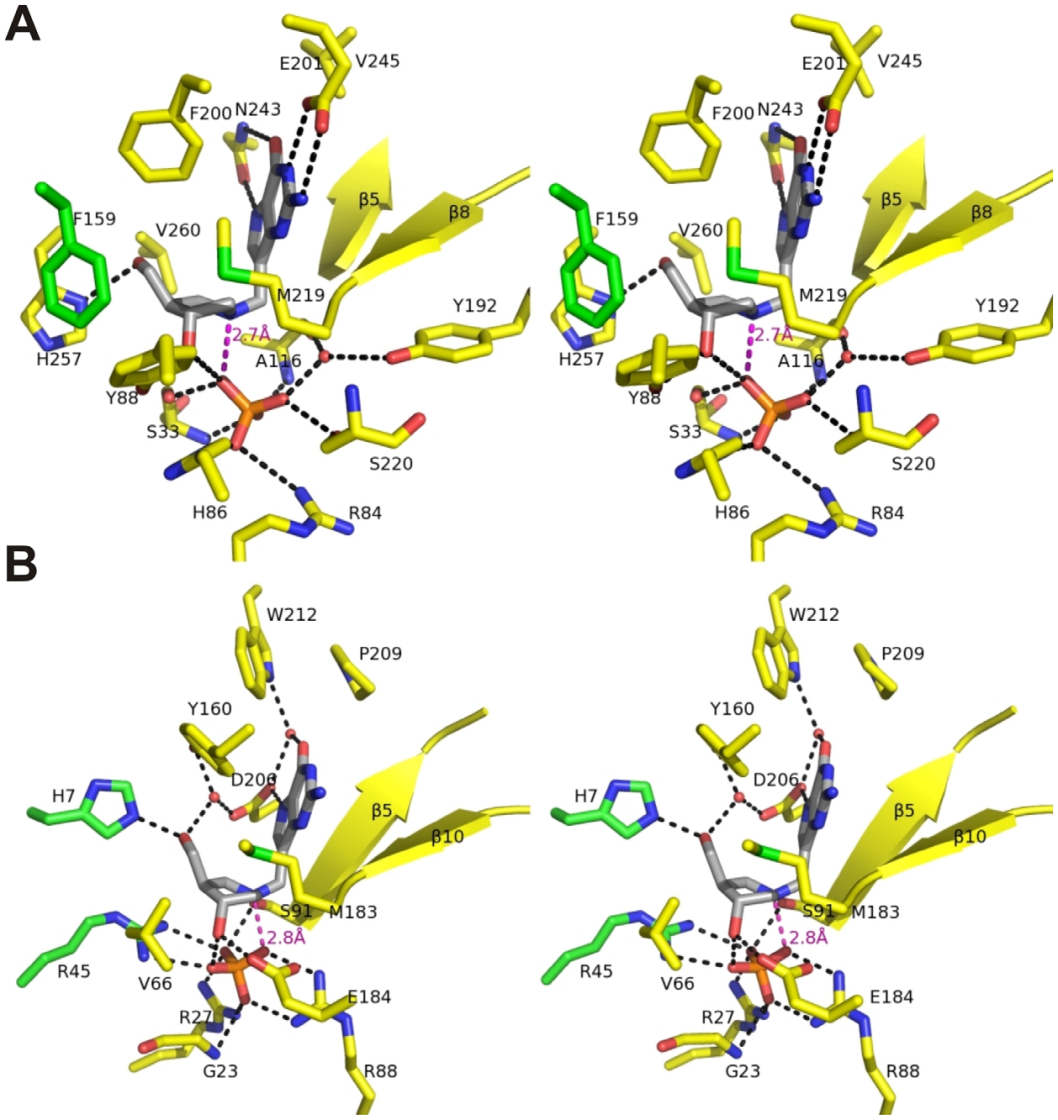
Stereo view crystal structure of BCX4945 and phosphate bound to hPNP and *Pf*PNP. Crystal structures of hPNP-BCX4945-PO_4_ (**A**) and *Pf*PNP-BCX4945-PO_4_ (**B**) were determined to 2.3 and 2.0 Å resolution, respectively. BCX4945 (grey) active site residues (yellow), residues from adjacent subunits (green) and phosphate (orange) are indicated. Hydrogen bonds are indicated as black dashed lines. The ion-pair interaction between the ribocation mimic and phosphate is indicated as a magenta dashed line with the distances indicated.

Crystal structures of human and *P. falciparum* PNPs bound to BCX4945 were determined to 2.3 and 2.0 Å resolution, respectively (Table S2). Other structures of human and malarial PNPs have been reported, but these are the first with BCX4945 [28], [29] (Figure S6 and S7). Human and malarial PNPs are structurally diverse but both catalyze the phosphorolysis of purine nucleosides using the same D_N_*A_N_ (S_N_1) stepwise mechanism [18]. Human and malarial PNPs have similar geometric arrangement of the purine-, ribose- and phosphate-binding sites [29] but use different amino acids within the catalytic sites and exhibit distinct kinetic properties (Figure 4, Table S3) [29], [30].

Human PNP holds the 9-deazaguanine moiety of BCX4945 in a hydrophobic pocket with specific interactions to Asn243 and Glu201 as hydrogen acceptors from the NH7 (2.8 Å), NH1 (2.8 Å) and NH2 (3.0 Å) groups (Figure 4A). Asn243 also donates a hydrogen bond to O6 of the deazaguanine group (3.1 Å). The dihydroxypyrrolidine cation of BCX4945 serves as a transition state ribocation mimic and is surrounded by hydrophobic residues (Tyr88, Phe159, Phe200, Met219) with a H-bond between the 5′-hydroxyl mimic and His257 (2.8 Å) and with Phe159 contributed from the adjacent subunit of the trimer (Figure 4A, Figure S6A). The nucleophilic phosphate anion forms a 2.7-Å ion pair with the cationic nitrogen of the ribocation mimic and hydrogen bonds to the 3′-hydroxyl group, which is additionally hydrogen-bonded to Tyr88 (2.6 Å) (Figure 4A). Phosphate bound to human PNP is dianionic [31] and its binding site includes amide nitrogens (Ser33, Ala116), the side chains of Ser33, Arg84, His86 and Ser220, and the cation contact from BCX4945. The carbonyl of Ala116 and the side chain of Tyr192 interact with phosphate through a structural water molecule (Figure 4A). The close ion pair between the tightly bound phosphate and BCX4945, in a hydrophobic environment, is a major contributor to its picomolar binding affinity.

The purine binding site of *Pf*PNP is surrounded by peptide bonds (Ser91, Cys92 and Gly93 of the β5 strand and Val181, Glu182 and Met183 of the β10 strand) and hydrophobic residues (Tyr160, Met183, Pro209 and Trp212). The hydrogen bond between Asp206 and NH7 (2.8 Å) mimics the interaction involving the N7-protonated guanine leaving group at the transition state. A structural water molecule stabilized by Asp206 and Trp212 is in a hydrogen bond with O6 (2.7 Å) of BCX4945 (Figure 4B). The ribocation mimic of BCX4945 is surrounded by His7, Val66, Tyr160, Met183, Glu184 and Asp206, with His7 contributed from the adjacent subunit of the hexamer (Figure 4B, Figure S6B). The 3′- and 5′-hydroxyl groups of BCX4945 form hydrogen bonds with side chains of Glu184 (2.6 Å) and His7 (2.6 Å). The side chains of Tyr160 (2.8 Å) and Asp206 (2.8 Å) immobilize a water molecule in contact with the 5′-hydroxyl group (2.8 Å). Phosphate binding involves the amide nitrogens of Gly23 and side chains of Arg27, Arg45, Arg88 and Ser91, with Arg45 contributed from the adjacent subunit (Figure 4B). Similar to human PNP, a 2.8-Å ion pair between phosphate and the ribocation mimic is a favorable energetic feature of BCX4945 binding. Phosphate also interacts with the ribocation mimic via the 3′-hydroxyl of BCX4945 (Fig. 4B).

## Discussion

BCX4945, a transition state analogue of both human and malarial PNPs, cleared *P. falciparum* infection in *Aotus* non-human primates, a close model of human malaria infection. Monkeys cleared of blood parasites by oral BCX4945 treatment remained negative for several days followed by recrudescence with a decreased rate of parasitic growth. This pattern in the *Aotus* model is termed ‘clearance and recrudescence’ (see materials and methods) and is often seen with effective antimalarials (see below). Parasite killing results from “purine starvation” since added hypoxanthine rescues cultured parasites from PNP inhibitors. Although metabolic studies also indicate inhibition of polyamine synthesis, as expected from the dual function of PNP in *P. falciparum*, polyamines are reduced, not depleted, indicating purine starvation as the dominant mechanism of action.

Inhibition of PNP as an antimalarial approach differs from all current therapies, suggesting utility in extending the current WHO protocols for malaria treatment using combination therapy approaches. The chemical class of PNP transition state analogues that includes BCX4945 is reported to be orally available and to exhibit low toxicity in humans [17], [32], [33]. BCX4945 showed a moderate oral availability (*F* = 28%) in *Aotus* monkeys. Our metabolic and structural studies support BCX4945 action via its tight binding to the PNP targets to induce purine-less death in *P. falciparum* both *in vitro* and *in vivo*. PNP is abundant in mammalian erythrocytes; and erythrocytes are constantly replaced. Therefore, the purine levels in *Aotus* blood returned to normal after treatment ceased, even when blood PNP remained partially inhibited.

Pharmacokinetics of BCX4945 indicated rapid clearance from *Aotus* plasma (t ½ = 1.2–1.3 h) and was similar in infected animals. However, the *C*_max_ value for BCX4945 was achieved earlier in infected-monkeys (2 h versus 6 h in uninfected-monkeys). BCX4945 absorption is reminiscent of quinine metabolism where acute malaria influences its absorption [34]. A short half-life in plasma is also observed in artemisinin derivatives (<3 h), which are current front-line antimalarials [35]. Artemisinin derivatives are not well suited for monotherapy as they also cause clearance and recrudescence [36], [37]. However, the WHO has initiated artemisinin-based combination therapy with great success [35].

In our studies with BCX4945 given once a day (50 mg kg^−1^) for three days, *P. falciparum* parasitaemia was reduced by >99% (Figure S5A). However, with once-a-day dosing, BCX4945 concentration in plasma fell to zero between doses. When infected *Aotus* monkeys were treated twice a day (50 mg kg^−1^) for seven days, this dosing schedule cleared parasitaemia to >99.99% of the original infection but recrudescence occurred after a period of days. Increasing the frequency of BCX4945 administration was more effective since the concentration of BCX4945 remained over 500 nM in plasma at all times during the treatment period (Figure S3). The presence of BCX4945 in plasma is critical for its efficacy.

Metabolic differences between humans and *Aotus* suggest PNP inhibition as anti-malaria therapy may be more effective in humans. *Aotus* blood contains hypoxanthine at concentrations an order of magnitude greater than found in humans and hypoxanthine is the limiting metabolite for purine salvage in the parasite. This metabolic finding suggests that BCX4945 treatment may be more effective to reduce blood hypoxanthine in humans than in *Aotus*. Since the mechanism of action of BCX4945 differs from all other antimalarials currently in human use, its potential application in combination therapies, similar to the use of artemisinin-derivatives, is attractive.

Our studies using *Aotus-P. falciparum* model indicate that similar to artemisinin [35], the clearance and recrudescence pattern may result from a combination of the short drug half-life in plasma and the high levels of hypoxanthine found in *Aotus* monkeys. However, further investigations are required to establish potential therapeutic resistance.

This work reports a promising agent to block the purine salvage and inhibit polyamine pathways in lethal infections of *P. falciparum*. The oral availability, chemical stability, unique mechanism of action and low toxicity to humans makes PNP transition state-analogues novel candidates for the treatment of malaria in combination therapies.

## Materials and Methods

### Ethics statement

Pharmacokinetic and efficacy of BCX4945 in *Aotus* monkeys infected with *P. falciparum* were performed under the Institutional Animal Care and Use Committee of the Albert Einstein College of Medicine acting under NIH/OLWA guidelines, Protocol Title: Efficacy and Pharmacokinetics of Immucillin in the *Aotus Plasmodium falciparum* Model, Protocol No. 20080706, approval date October 20, 2008. This Institutional Animal Welfare Assurance (A3312-01) is fully accredited by the Association for the Assessment and Accreditation of Laboratory Animal Care (AAALAC), February 22, 1983. Animal research at the Gorgas Research Institute was approved by the ICGES Institutional Animal Care and Use Committee (IACUC) (CIUCAL) under the accession number 2008/03, reviewed and approved on July 2, 2008. All protocols were approved under the International Guiding Principles for Biomedical Research Involving Animals developed by the Council for International Organizations of Medical Sciences (CIOMS), and the laws of the Republic of Panama. The Statement of Compliance at the Gorgas Research Institute (Instituto Commemorativo Gorgas de Estudios de la Salud), Panama, has been reviewed by the Department of Health & Human Services, Public Health Service, National Institutes of Health, where the statement of Compliance (Assurance) with standards for Humane Care and Use of Laboratory Animals was reviewed by the Office for Protection from Research Risks (OPRR) and approved under approval number #A5389-01, originally approved on March 9, 1999. Animals used under these approvals are documented third-generation laboratory-bred animals. They are housed under conditions permitting social interactions, laboratory breeding and family interactions. All infections and blood sample collections were conducted under protocols that ameliorate suffering in accordance with the recommendations of the Weatherall report, “The use of non-human primates in research”. Transfer of blood samples from Panama to New York was accomplished under permit numbers 2009-08-46 issued August 12, 2009 and 2010-11-080 issued November 22, 2010 by the Department of HHS, Center for Disease Control, Atlanta in a ‘Permit to Import or Transfer Etiological Agents or Vectors of Human Disease’. Sample shipping was also approved by the Commission on International Trade in Endangered Species (CITES), Panama, permit number SEX/A-19-10. Human erythrocytes were obtained from healthy donors under the Albert Einstein College of Medicine Committee on Clinical Investigations Protocol M-1063.

### Inhibition tests

The effect of BCX4945 on parasite growth of 3D7, Dd2 and FVO strains was measured in a 72 h growth assay in the presence of drug as described [10]. Briefly, synchronous culture in the schizont stage was cultured for 24 h in media containing 10 µM of hypoxanthine. Ring stage parasite cultures (200 µl per well, with 1% hematocrit and 1% parasitaemia) were then grown for 72 h in the presence of increasing concentrations of the drug. After 72 h in culture, parasite viability was determined by DNA quantitation using PicoGreen (Invitrogen) as described by Quashie and colleagues [20]. The half-maximum inhibitory concentration (IC_50_) calculation was performed with Origin software (OriginLab) using a nonlinear regression curve fitting. Half-maximum inhibitory concentration (IC_50_) is the concentration of inhibitor needed to kill 50% of parasites.

### Inosine and hypoxanthine rescue assay

Synchronized, schizont stage parasites were washed in purine-free medium to remove excess hypoxanthine and inoculated into freshly drawn erythrocytes that had been washed and resuspended in purine-free medium. The schizont stage parasites were cultured in purine-free medium for 24 h to further reduce hypoxanthine inside the cell. The resulting ring-stage parasites (1% parasitaemia, 4% hematocrit) were incubated with 30 µM of BCX4945 for 30 min at 37 °C. After incubation, parasites without washing the excess of inhibitor were diluted into 96-well microtiter plates to a final BCX4945 concentration of 15 µM, 2% hematocrit, 1% parasitaemia, increasing hypoxanthine or inosine concentrations up to 200 µM and cultured for 72 h. Parasite viability was determined by DNA quantitation as described by Quashie and colleagues [20]. For each condition, two independent experiments were carried out in triplicate.

### Metabolic labeling and HPLC analysis

Infected-erythrocytes in trophozoite and schizont stages treated with 10 µM BCX4945 or untreated were metabolically labeled with 1 µM of [2,8-^3^H]inosine (50 Ci/mmol, Moravek) or [2,8-^3^H]hypoxanthine (30 Ci/mmol, Moravek) at 37 °C for 1 h. Samples were extracted with perchloric acid (PCA), neutralized and analyzed by HPLC as described [10].

### Polyamine quantification

Polyamines from 0.5 M PCA extracts were analyzed as the dansyl-derivatives by HPLC/fluorescence on a Waters Millennium system [38]. Protein concentration was determined with a bicinchoninic acid (BCA) protein assay kit according to the manufacturer's instructions (Pierce) using BSA as a standard. Data from biological samples were normalized to the protein content.

### Purine metabolites and BCX4945 quantitation

Samples from blood cells, plasma and media were extracted and analyzed in duplicate. Samples were placed in 96 deep-well plates, treated with 0.5 M HClO_4_ at 1∶7 (v/v, sample/HClO_4_) and immediately mixed with 7 pmol of each internal standard, [2, 8-^2^H_2_]adenosine, [2, 8-^2^H_2_]inosine, [2, 8-^2^H_2_]hypoxanthine and [*methylene*-^2^H_2_]BCX4945 synthesized as described below. Samples were incubated for 20 min at 4 °C and neutralized with 5 M KOH at 10∶1 (v/v, HClO_4_/KOH) for 20 min at 4 °C. Plates were centrifuged (10 min at 4,000 rpm, 4°C) and supernatants were filtered through a MultiScreen® Filter Plate with Ultracel®-10 Membrane (Millipore). Metabolite and inhibitor levels were quantified by UPLC/MS/MS using a Xevo TQ mass spectrometer (Waters). The separation of adenosine, inosine, hypoxanthine, 2′-deoxyguanosine, MTA, MTI and BCX4945 was achieved with an Acquity HSS T3 column (2.1×100 mm, 1.8 µm, Waters) at 60 °C. The eluent system was composed of 5 mM ammonium formate in water (A) and 5 mM ammonium formate in methanol (B) with a gradient of 98% eluent A to 30% eluent B from 0.1 to 1 min, 70% eluent A to 80% eluent B from 1 to 1.5 min and back to 98% eluent A from 1.5 to 2 min at a flow rate of 0.6 ml min^−1^. Quantitative determination was performed in ESI positive-ion mode using multiple-reaction monitoring (MRM) mode. The ion transitions, cone voltage and collision energy used for ESI-MS/MS analysis are presented in Table S4. The ESI capillary voltage was 0.3 kV, source temperature was set at 150 °C and desolvation temperature at 450 °C. Data acquisition and analysis were carried out by MassLynx V4.1 and QuanLynx software (Waters). The concentration of the metabolites was calculated by interpolation of the observed analyte/internal standard peak-area ratio with the corresponding calibration curve.

### Pharmacokinetic and efficacy studies

Laboratory bred *Aotus lemurinus lemurinus* monkeys (0.75–0.89 kg) were maintained as described [39]. Individuals received orally 50 mg kg^−1^ of BCX4945 in 10% sucrose by gastric intubation or intravenously 10 mg kg^−1^ in phosphate buffered saline solution (pH 7.4), once a day for three days. Blood samples were collected before the treatment started (t = 0), 0.05 h, 0.5 h, 1 h, 2 h, 6 h, 24 h, 48 h, and 6, 12 and 18 days after the first dose from each monkey and processed as described below. Pharmacokinetic parameters were calculated using PKSolver [40]. For efficacy studies, *Aotus* monkeys were inoculated intravenously with 5×10^6^ parasites of the FVO strain of *P. falciparum* in an approved treatment protocol. Four days after the onset of patency (parasite counts ∼5.0×10^3^/µl blood), monkeys received orally 50 mg kg^−1^ of BCX4945 in 10% sucrose, once a day for three days or twice a day for seven days. Parasitaemia was determined by Giemsa-stained thick blood smears prepared daily from the marginal ear vein [41]. Blood samples were collected from each monkey and transferred to tubes containing lithium heparin, centrifuged and both plasma and blood cells were frozen at −80 °C until analysis. Animals received a physical examination and blood chemistry profiles were monitored after treatment as described previously [42]. Any monkey not responding appropriately to treatment was cured of its infection with mefloquine (40 mg kg^−1^). Response to treatment is categorized as clearance and cure, clearance and recrudescence, or suppression without clearance. The day of clearance is defined as the first of three consecutive days in which the thick blood films are parasite negative. The day of recrudescence is the first of three consecutive days of positive thick blood films after a period of clearance. Suppression is defined as a transient decrease in the parasite count post-treatment without clearance.

### Blood PNP activity

Blood cell pellets were diluted (1∶1) in PBS containing 0.3% Triton X-100. PNP activity was determined spectrophotometrically by adding 1 µl of the cell lysate to 1 ml of the reaction buffer containing 50 mM KH_2_PO_4_ (pH 7.5), 1 mM inosine and 20 mU xanthine oxidase (Sigma). Reaction progress was monitored at 293 nm for 20 min at 25 °C. After the reaction was completed, sample hemoglobin concentration was determined by measuring absorption at 414 nm and PNP activity was normalized to hemoglobin concentration.

### Crystallography

Recombinant hPNP and *Pf*PNP proteins were expressed and purified as described before [29], [43]. The hPNP protein was prepared in 50 mM Tris-HCl buffer at pH 8.5 containing 1 mM dithiothreitol (DTT). The final concentration for crystallization was 12 mg ml^−1^ in the presence of 1 mM BCX4945 and 5 mM potassium phosphate; crystals were grown by sitting-drop vapour diffusion at room temperature using 25% PEG 3350, 100 mM Tris-HCl (pH 8.5) and 200 mM ammonium sulfate as the precipitant. The *Pf*PNP protein was prepared in 50 mM HEPES (pH 7.4), 1 mM DTT and 3 mM potassium phosphate. The final concentration for crystallization was 10 mg ml^−1^ in the presence of 0.7 mM BCX4945. The *Pf*PNP-BCX4945 complex was crystallized in 10% PEG3350 and 100 mM sodium formate using sitting-drop vapour diffusion at room temperature. Crystals of hPNP-BCX4945 were transferred into a fresh drop of the crystallization solution supplemented with 20% glycerol and flash-cooled in liquid nitrogen. Crystals of *Pf*PNP-BCX4945 were transferred to a reservoir solution supplemented with 20% 2-methyl-2,4-pentanediol (MPD) prior to flash-cooling in liquid nitrogen. X-ray diffraction data were collected at Beamline X29A at Brookhaven National Laboratory. All data were processed with the HKL2000 program suite and the data processing statistics are presented in Table S2. The crystal structures of hPNP-BCX4945 and *Pf*PNP-BCX4945 were determined by molecular replacement in MOLREP using the published structures of hPNP (PDB accession 3K8O) and *Pf*PNP (PDB accession 1Q1G), respectively, as search models [44]. The models without BCX4945 were first rebuilt in COOT and refined in Refmac5 [45], [46]. The BCX4945 molecule was added in the last stages of refinement using the *F_o_−F_c_* map and refined in Refmac5 [46]. The refinement statistics are summarized in Table S2. The BCX4945-omitted electron density maps of the hPNP-BCX4945 and *Pf*PNP-BCX4945 complexes are shown in Figure S7. Atomic coordinates and structure factors for the crystal structures have been deposited with the Protein Data Bank under accession codes 3PHB for human PNP with bound BCX4945 and phosphate and 3PHC for *Pf*PNP with bound BCX4945 and phosphate.

### Synthesis of deuterated internal standards

#### Preparation of [2,8-^2^H_2_]hypoxanthine and [2,8-^2^H_2_]adenosine

Deuterium was incorporated into the purine rings of hypoxanthine and adenosine following slight modification of the procedure of Sajiki *et al* [47]. Hypoxanthine or adenosine (1 mmol) was dissolved in 10 ml of D_2_O (99.8% D) in a round-bottom flask equipped with a side arm attached through a stopcock to a vacuum source. After adding 10% Pd/C (10% w/w), a septum-topped condenser was affixed and the reaction flask was equilibrated with hydrogen gas by repeated evacuation and addition of hydrogen from a needle-tipped balloon. The mixture was refluxed for 24 h, and the Pd/C was filtered off by passage through a 0.45-µm syringe filter. Rotary evaporation of the filtrate yielded >90% of a white solid. ESI-MS analysis (Finnigan LCQ, positive mode) revealed the following isotopic distribution: hypoxanthine, *m*/*z* 139 (^2^H_2_-M+H^+^, 95%), 138 (^2^H_1_-M+H^+^, 5%); adenosine, *m*/*z* 270 (^2^H_2_-M+H^+^, 95%), 269 (^2^H_1_-M+H^+^, 5%).

#### Preparation of [2,8-^2^H_2_]inosine

[2,8-^2^H**_2_**]Adenosine (149 mg, 0.553 mmol) was dissolved in 10 ml distilled water by warming briefly in a microwave oven. Upon cooling to room temperature, 15 units of adenosine deaminase (Sigma, Type IX, calf spleen) was added, and the reaction mixture was left for 12 h. Formation of inosine was confirmed by UV-vis spectroscopy (λ_max_ = 251 nm). Inosine was isolated by reversed-phase HPLC using a Waters 600 system with a 486 detector (monitoring at 260 nm) and a Waters Xterra Prep C18 column (30×250 mm). Elution was achieved isocratically with 90% A (distilled water):10% B (50% aqueous methanol) at 14 ml min^−1^, yielding 110 mg (81%) of inosine (*t*_R_ = 8 min) and ∼1 mg of unreacted adenosine (*t*_R_ = 15 min). ESI-MS analysis of inosine revealed *m*/*z* 271 (^2^H_2_-M+H^+^, 95%), 270 (^2^H_1_-M+H^+^, 5%).

#### Preparation of [*methylene*-^2^H_2_]BCX4945

[*methylene*-^2^H_2_]BCX4945 was prepared by Mannich reaction according to the previously reported procedure [48] with slight modification. To a solution of (3*R*,4*R*)-3-hydroxy-4-(hydroxymethyl)pyrrolidine hydrochloride [49] (15 mg, 0.1 mmol), sodium acetate (8.2 mg, 0.1 mmol), and [^2^H_2_]formaldehyde (Cambridge Isotope Laboratories, 16 mg of 20% w/w aqueous solution, 0.1 mmol) in 0.5 ml H_2_O was added 9-deazaguanine (15 mg, 0.1 mmol), and the mixture was heated to 95°C for 21 h. The title compound was purified as the hydrochloride salt by preparative HPLC using isocratic 90% A (50 mM ammonium formate, adjusted to pH 4.0 with formic acid):10% B (50% aqueous methanol) at 14 ml min^−1^, yielding 20 mg (70%) of a yellowish glass. HPLC retention time (*t*_R_ = 5 min) agreed with that of an unlabeled BCX4945 standard. ESI-MS analysis revealed *m*/*z* 282 (^2^H_2_-M+H^+^, 100%).

## Supporting Information

Figure S1 **Hypoxanthine/inosine rescue assays.** Inosine and hypoxanthine supplementation and recovery of growth analysis in *P. falciparum* cultures treated with 15 µM BCX4945. Infected erythrocytes were cultured in the absence of exogenous purines followed by incubation in the presence of the BCX4945 and the indicated concentrations of purine for 72 h, followed by DNA quantitation. Percent parasite survival is (DNA synthesized by treated cells/DNA synthesized by control cells)×100. Means ± s.d. from 2 independent experiments are represented.
(TIF)

Figure S2 **PNP activity in uninfected and infected treated monkeys.** *Aotus* were treated with BCX4945 once a day for three days (50 mg kg^−1^). Blood PNP activity was assayed from an uninfected treated monkey (UTM, *n* = 1) and infected treated monkeys (ITMs, *n* = 3, means ± s.d.). Each sample was measured in triplicate. The PNP activity of infected-animals recovered faster after treatment stopped due to increased hematopoiesis in response to parasite-induced anemia. Similar values of PNP activity and recovery of activity after treatment were observed in all uninfected treated monkeys independent of the number of doses and the route of administration (data not shown).
(TIF)

Figure S3 **Purines and BCX4945 levels in plasma and blood cells from** ***P. falciparum*** **infected-** ***Aotus*** **monkeys untreated or orally treated with BCX4945 twice a day for seven days.** Samples from blood cells and plasma were extracted and analyzed in duplicate by UPLC-MS/MS. The concentration of the metabolites was calculated by interpolation of the observed analyte/internal standard peak-area ratio with the corresponding calibration curve. Time point (−4) indicates blood was taken before the monkey was infected. Time point (0) indicates that blood was drawn before the treatment started. Day 1 reflects metabolite and BCX4945 levels 24 h after the first dose and the metabolic effect of two BCX4945 doses within 24 h (also for days 2 to 5). Days 12 and 18 are counted from the start of treatment. Data are from infected untreated monkey (*n* = 1) and infected treated monkeys (*n* = 3, means ± s.d.).
(TIF)

Figure S4 **Single dose oral and intravenous pharmacokinetic analysis of BCX4945 in** ***Aotus*** **.** Uninfected (**A, B**) and *P. falciparum*-infected monkeys (**C, D**) were treated once orally (50 mg kg^−1^, filled circles, *n* = 3) or intravenously (IV, 10 mg kg^−1^, open squares, *n* = 4). BCX4945 levels in plasma and blood cells were measured by UPLC-MS/MS using [*methylene*-^2^H_2_]BCX4945 as an internal standard. Pharmacokinetic parameters were calculated using PKSolver.
(TIF)

Figure S5 **Three-day oral treatment of** ***P. falciparum-*** **infected** ***Aotus*** **and PNP activity.** (**A**) Parasitaemia in infected untreated monkey (IUM, *n* = 1) and infected treated monkeys (ITM, *n* = 3) with BCX4945. Grey bar on the x-axis indicates days of treatment. Oral once-a-day dosing (50 mg kg^−1^) for three days reduced parasitaemia by 99%. Parasite regrowth resumed after the last dose due to rapid BCX4945 clearance from plasma (see Figure S4). At day 8 post-infection, the control animal (IUM) was treated with one dose of mefloquine. Other animals were treated with one dose of mefloquine at day 10 (ITM1 and ITM2) and 12 (ITM3) post-infection. (**B**) Hypoxanthine (white bars) and inosine (black bars) concentration in uninfected- and *P. falciparum-*infected monkeys treated with oral BCX4945 for three days. One uninfected animal and three infected animals were treated (means ± s.d. are represented). *Plasmodium* infection reduces purine levels in plasma and the blood cells. Hypoxanthine (∼30 µM) reappeared in the blood of infected animals three days after the treatment ended.
(TIF)

Figure S6 **The crystal structure of hPNP and** ***Pf*** **PNP bound to BCX4945.** Crystal structures of hPNP-BCX4945-PO_4_ (**A**) and *Pf*PNP-BCX4945-PO_4_ (**B**) were determined to 2.3 and 2.0 Å resolution, respectively. Human PNP is a homotrimer with single-domain monomers of 10 β strands and 8 α helices. Monomer cores consist of a mixed seven-stranded β sheet (β2, β3, β4, β1, β5, β10 and β6) which is flanked by eight α-helices. β5 is extended and participates in an additional four-stranded β-sheet (β5, β9, β8 and β7). *Pf*PNP is a homohexamer where each monomer contains 11 β-strands and 7 α-helices and is folded similarly to hPNP monomers. Human and malarial PNP monomers share structural similarity (C_α_ r.m.s.d. of 2.5 Å) despite sharing only 14% sequence identity. The asymmetric unit of the crystal structure of hPNP-BCX4945-PO_4_ contained two distinct homotrimers while the asymmetric unit of crystal structure of *Pf*PNP-BCX4945-PO_4_ contained one homohexamer organized as a trimer of dimers. Despite differences in quaternary structure, the location of the active site within monomers is similar. Subunit contacts are essential to the formation of active sites in both PNPs. The active sites of hPNP are located at interacting subunits within the trimer, whereas the active sites of *Pf*PNP are face-to-face located at dimer interfaces.
(TIF)

Figure S7 **The BCX4945-omitted electron density maps of hPNP and** ***Pf*** **PNP bound to BCX4945.** (**A**) hPNP-BCX4945-PO_4_ and (**B**) *Pf*PNP-BCX4945-PO_4_. BCX4945 (grey), active site residues (yellow), residues from adjacent subunits (green) and phosphate (orange) are indicated. The BCX4945-omitted *mF_o_ - DF_c_* electron density map (contour at the 3 σ) is drawn in green. The partial BCX4945-omitted 2*mF_o_ - DF_c_* electron density map (contour at the 1 σ) is drawn in blue.
(TIF)

Table S1 Weights and complete blood chemistry profiles of *Aotus* monkeys.
(XLS)

Table S2 Data collection and refinement statistics.
(DOC)

Table S3 Residues in the active sites of human and *Pf*PNP.
(DOC)

Table S4 MRM acquisition settings for purines and BCX4945 quantitation.
(DOC)
