# Supplementary figures and images for: Plasmodium falciparum Parasites Are Killed by a Transition State Analogue of Purine Nucleoside Phosphorylase in a Primate Animal Model
Source: PLoS One. 2011 Nov 11;6(11):e26916. doi: 10.1371/journal.pone.0026916 (PMC3214022; doi:10.1371/journal.pone.0026916)

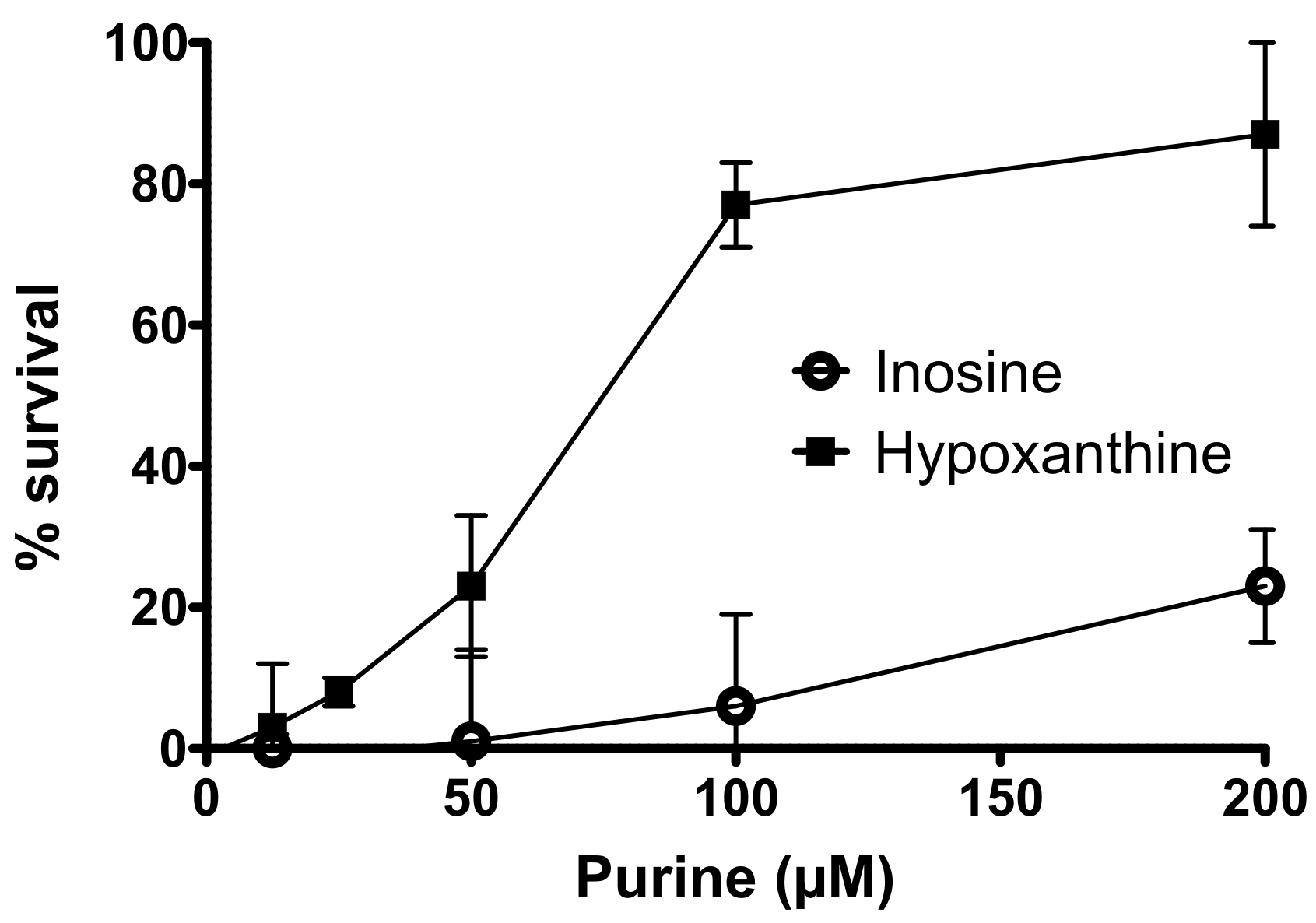

Supplement: Figure S1 — Hypoxanthine/inosine rescue assays. Inosine and hypoxanthine supplementation and recovery of growth analysis in P. falciparum cultures treated with 15 µM BCX4945. Infected erythrocytes were cultured in the absence of exogenous purines followed by incubation in the presence of the BCX4945 and the indicated concentrations of purine for 72 h, followed by DNA quantitation. Percent parasite survival is (DNA synthesized by treated cells/DNA synthesized by control cells)×100. Means ± s.d. from 2 independent experiments are represented. (TIF) [file pone.0026916.s001.tif]

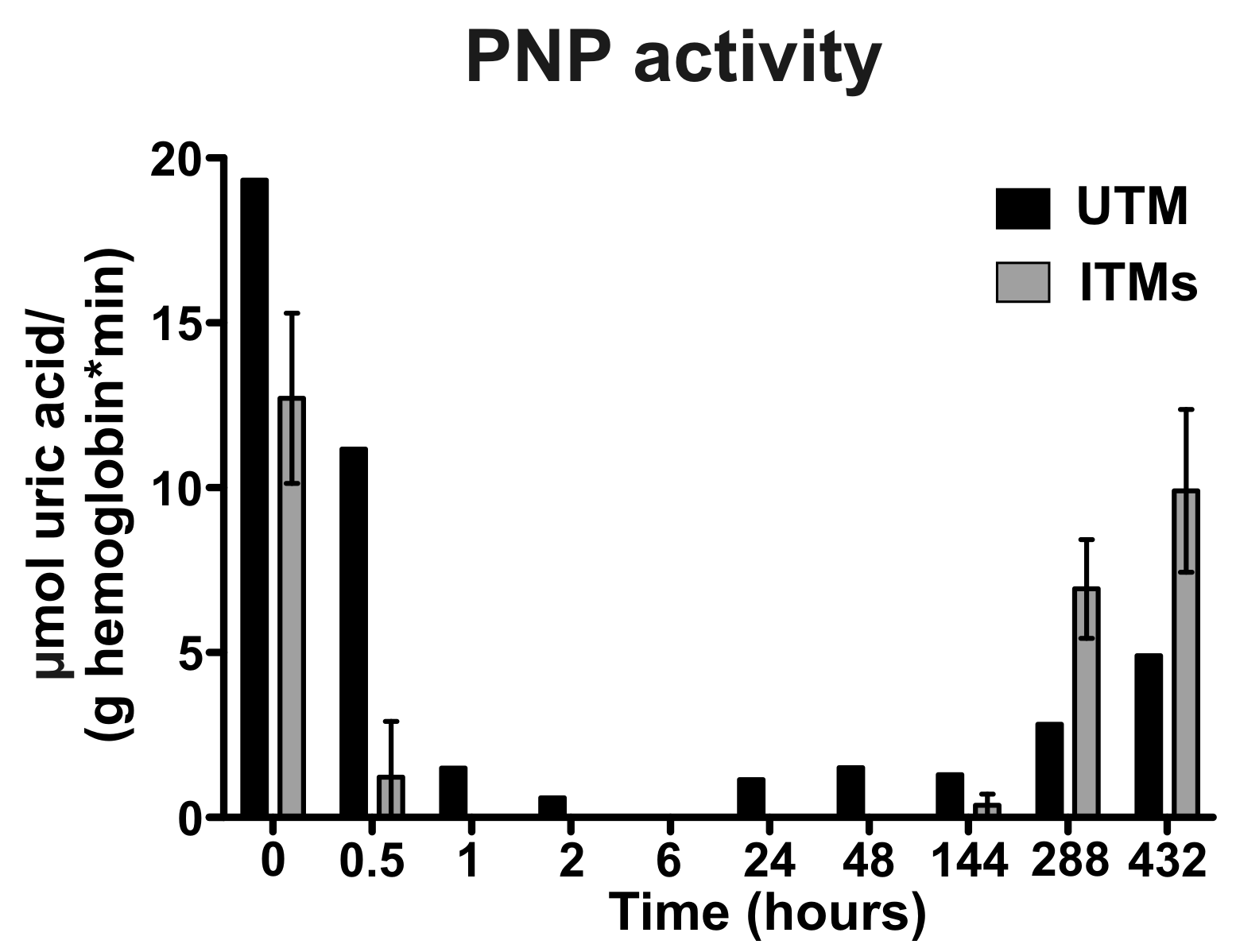

Supplement: Figure S2 — PNP activity in uninfected and infected treated monkeys. Aotus were treated with BCX4945 once a day for three days (50 mg kg−1). Blood PNP activity was assayed from an uninfected treated monkey (UTM, n = 1) and infected treated monkeys (ITMs, n = 3, means ± s.d.). Each sample was measured in triplicate. The PNP activity of infected-animals recovered faster after treatment stopped due to increased hematopoiesis in response to parasite-induced anemia. Similar values of PNP activity and recovery of activity after treatment were observed in all uninfected treated monkeys independent of the number of doses and the route of administration (data not shown). (TIF) [file pone.0026916.s002.tif]

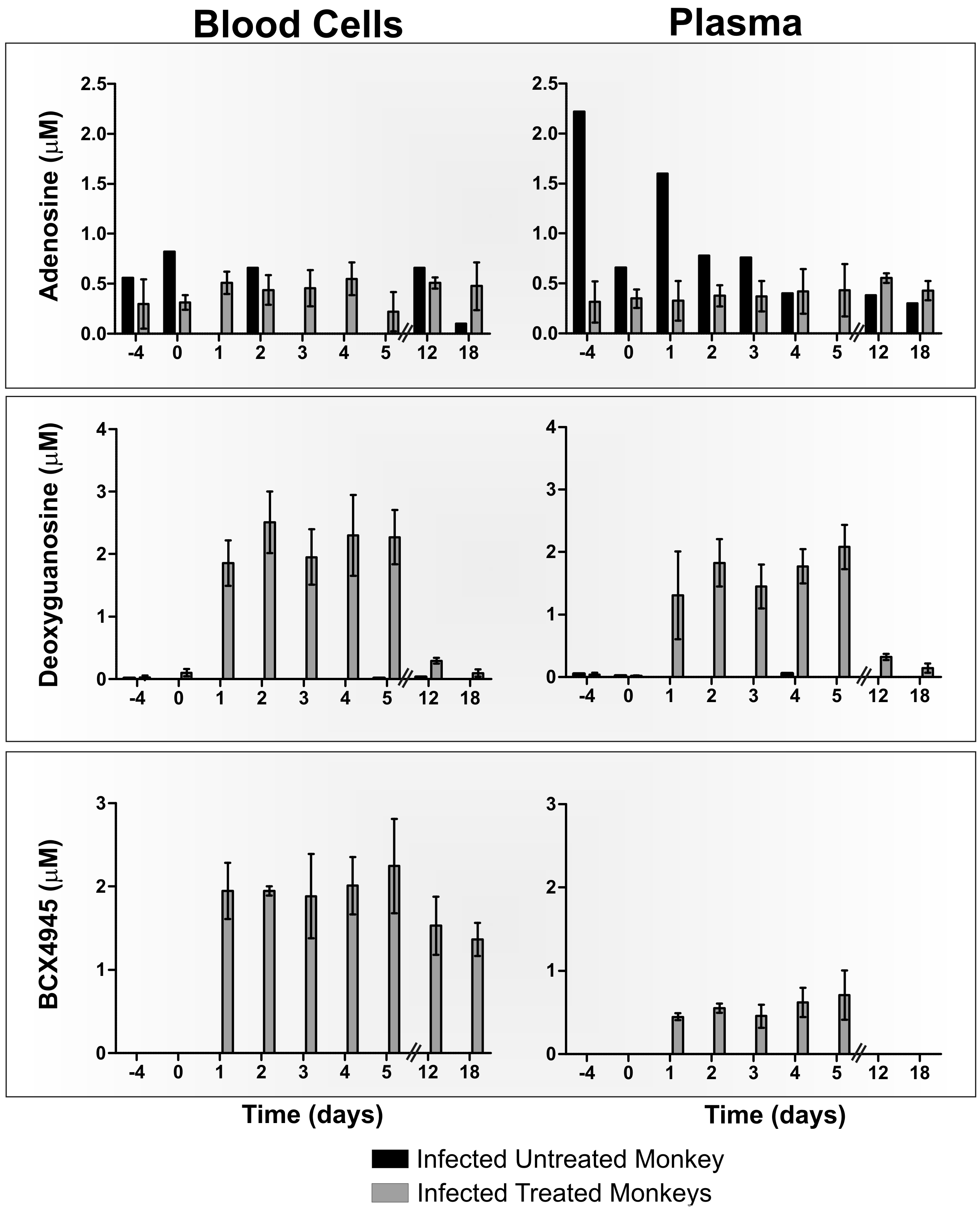

Supplement: Figure S3 — Purines and BCX4945 levels in plasma and blood cells from P. falciparum infected- Aotus monkeys untreated or orally treated with BCX4945 twice a day for seven days. Samples from blood cells and plasma were extracted and analyzed in duplicate by UPLC-MS/MS. The concentration of the metabolites was calculated by interpolation of the observed analyte/internal standard peak-area ratio with the corresponding calibration curve. Time point (−4) indicates blood was taken before the monkey was infected. Time point (0) indicates that blood was drawn before the treatment started. Day 1 reflects metabolite and BCX4945 levels 24 h after the first dose and the metabolic effect of two BCX4945 doses within 24 h (also for days 2 to 5). Days 12 and 18 are counted from the start of treatment. Data are from infected untreated monkey (n = 1) and infected treated monkeys (n = 3, means ± s.d.). (TIF) [file pone.0026916.s003.tif]

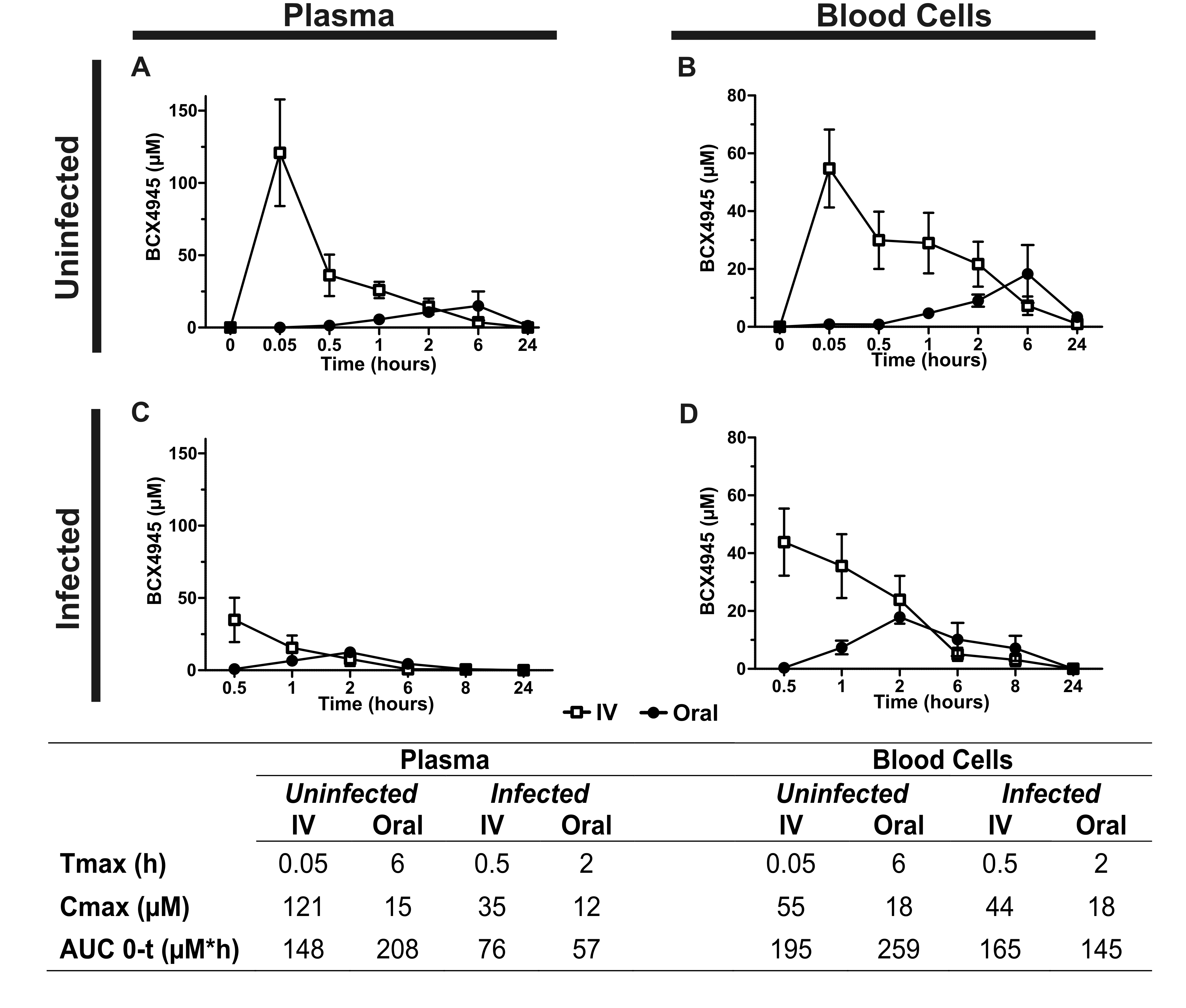

Supplement: Figure S4 — Single dose oral and intravenous pharmacokinetic analysis of BCX4945 in Aotus . Uninfected (A, B) and P. falciparum-infected monkeys (C, D) were treated once orally (50 mg kg−1, filled circles, n = 3) or intravenously (IV, 10 mg kg−1, open squares, n = 4). BCX4945 levels in plasma and blood cells were measured by UPLC-MS/MS using [methylene-2H2]BCX4945 as an internal standard. Pharmacokinetic parameters were calculated using PKSolver. (TIF) [file pone.0026916.s004.tif]

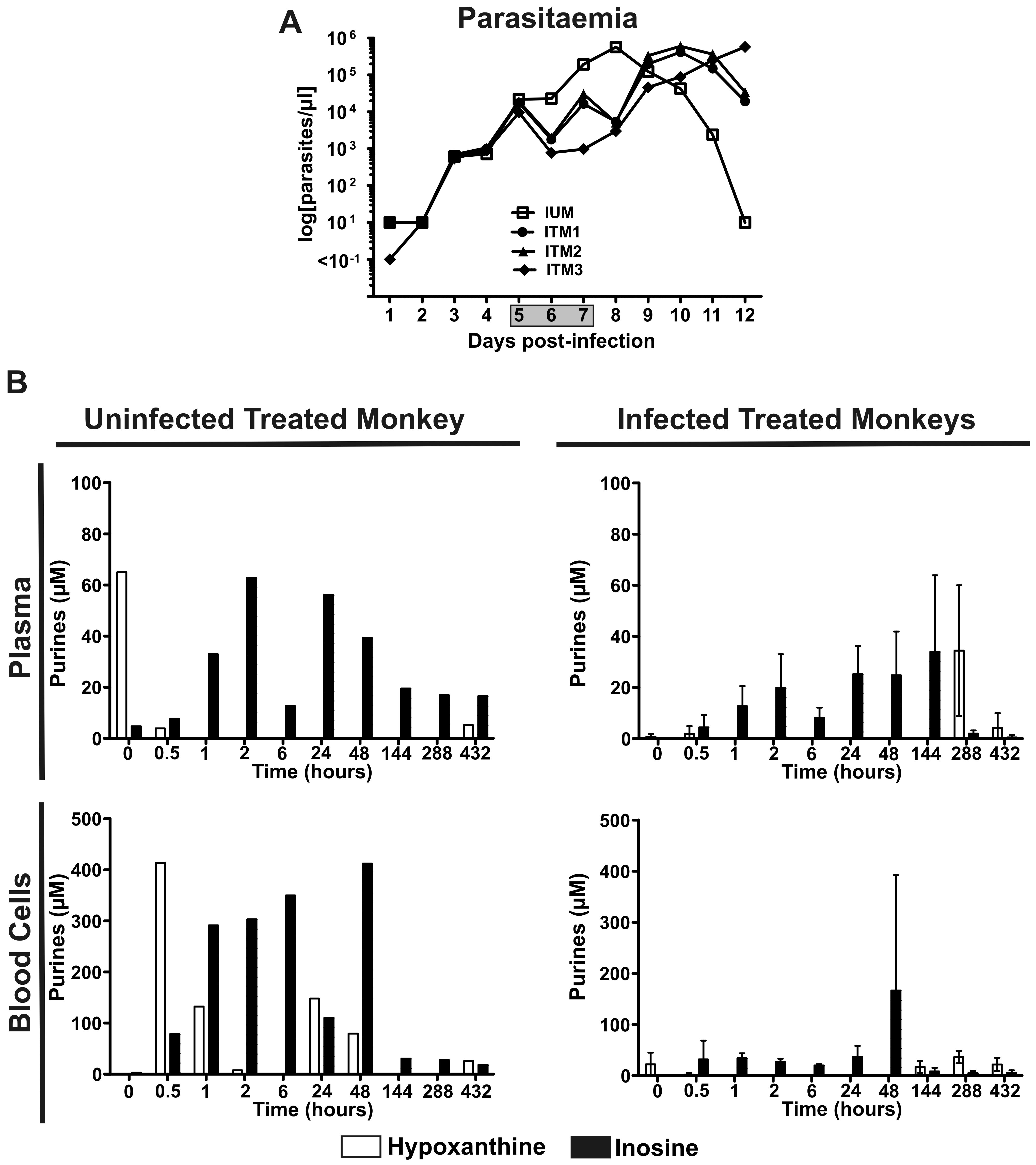

Supplement: Figure S5 — Three-day oral treatment of P. falciparum- infected Aotus and PNP activity. (A) Parasitaemia in infected untreated monkey (IUM, n = 1) and infected treated monkeys (ITM, n = 3) with BCX4945. Grey bar on the x-axis indicates days of treatment. Oral once-a-day dosing (50 mg kg−1) for three days reduced parasitaemia by 99%. Parasite regrowth resumed after the last dose due to rapid BCX4945 clearance from plasma (see Figure S4). At day 8 post-infection, the control animal (IUM) was treated with one dose of mefloquine. Other animals were treated with one dose of mefloquine at day 10 (ITM1 and ITM2) and 12 (ITM3) post-infection. (B) Hypoxanthine (white bars) and inosine (black bars) concentration in uninfected- and P. falciparum-infected monkeys treated with oral BCX4945 for three days. One uninfected animal and three infected animals were treated (means ± s.d. are represented). Plasmodium infection reduces purine levels in plasma and the blood cells. Hypoxanthine (∼30 µM) reappeared in the blood of infected animals three days after the treatment ended. (TIF) [file pone.0026916.s005.tif]

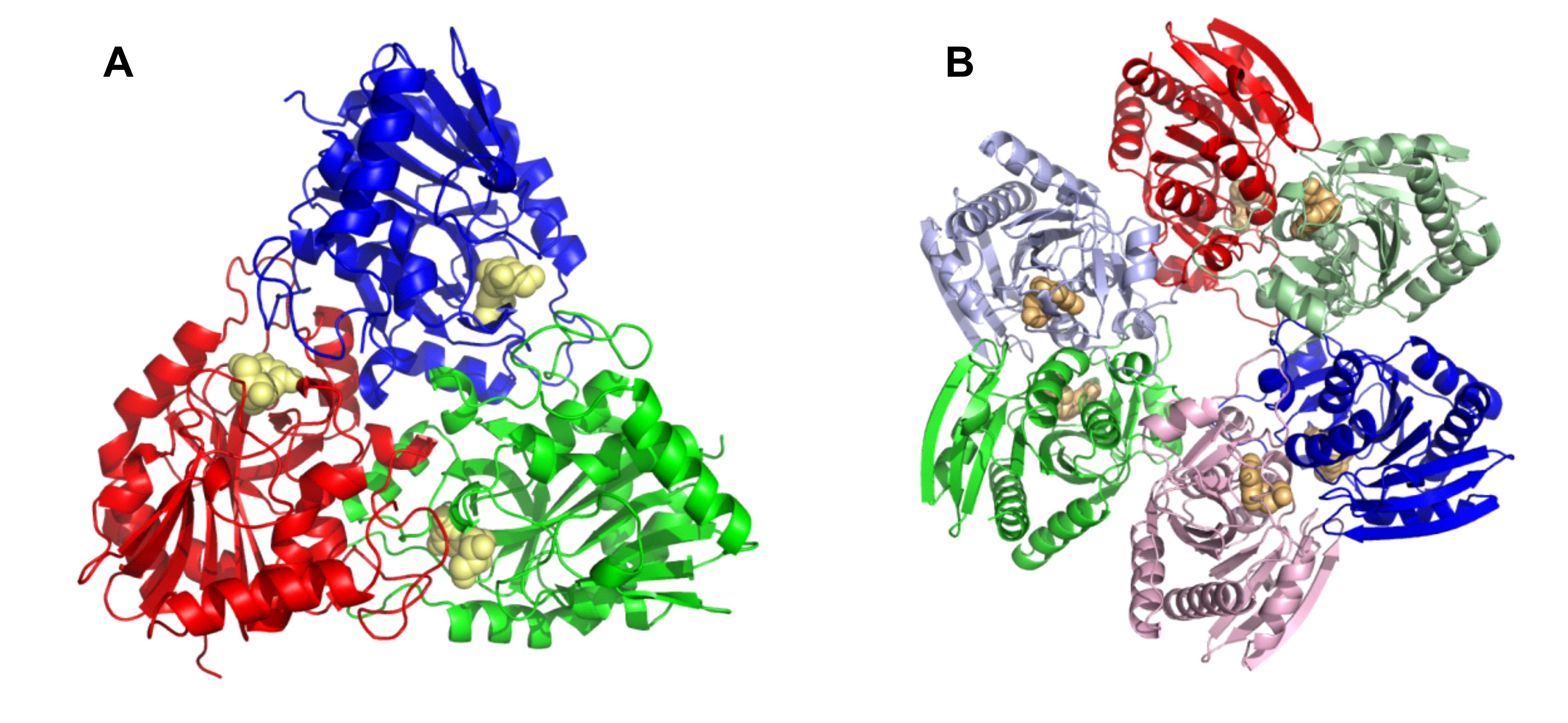

Supplement: Figure S6 — The crystal structure of hPNP and Pf PNP bound to BCX4945. Crystal structures of hPNP-BCX4945-PO4 (A) and PfPNP-BCX4945-PO4 (B) were determined to 2.3 and 2.0 Å resolution, respectively. Human PNP is a homotrimer with single-domain monomers of 10 β strands and 8 α helices. Monomer cores consist of a mixed seven-stranded β sheet (β2, β3, β4, β1, β5, β10 and β6) which is flanked by eight α-helices. β5 is extended and participates in an additional four-stranded β-sheet (β5, β9, β8 and β7). PfPNP is a homohexamer where each monomer contains 11 β-strands and 7 α-helices and is folded similarly to hPNP monomers. Human and malarial PNP monomers share structural similarity (Cα r.m.s.d. of 2.5 Å) despite sharing only 14% sequence identity. The asymmetric unit of the crystal structure of hPNP-BCX4945-PO4 contained two distinct homotrimers while the asymmetric unit of crystal structure of PfPNP-BCX4945-PO4 contained one homohexamer organized as a trimer of dimers. Despite differences in quaternary structure, the location of the active site within monomers is similar. Subunit contacts are essential to the formation of active sites in both PNPs. The active sites of hPNP are located at interacting subunits within the trimer, whereas the active sites of PfPNP are face-to-face located at dimer interfaces. (TIF) [file pone.0026916.s006.tif]

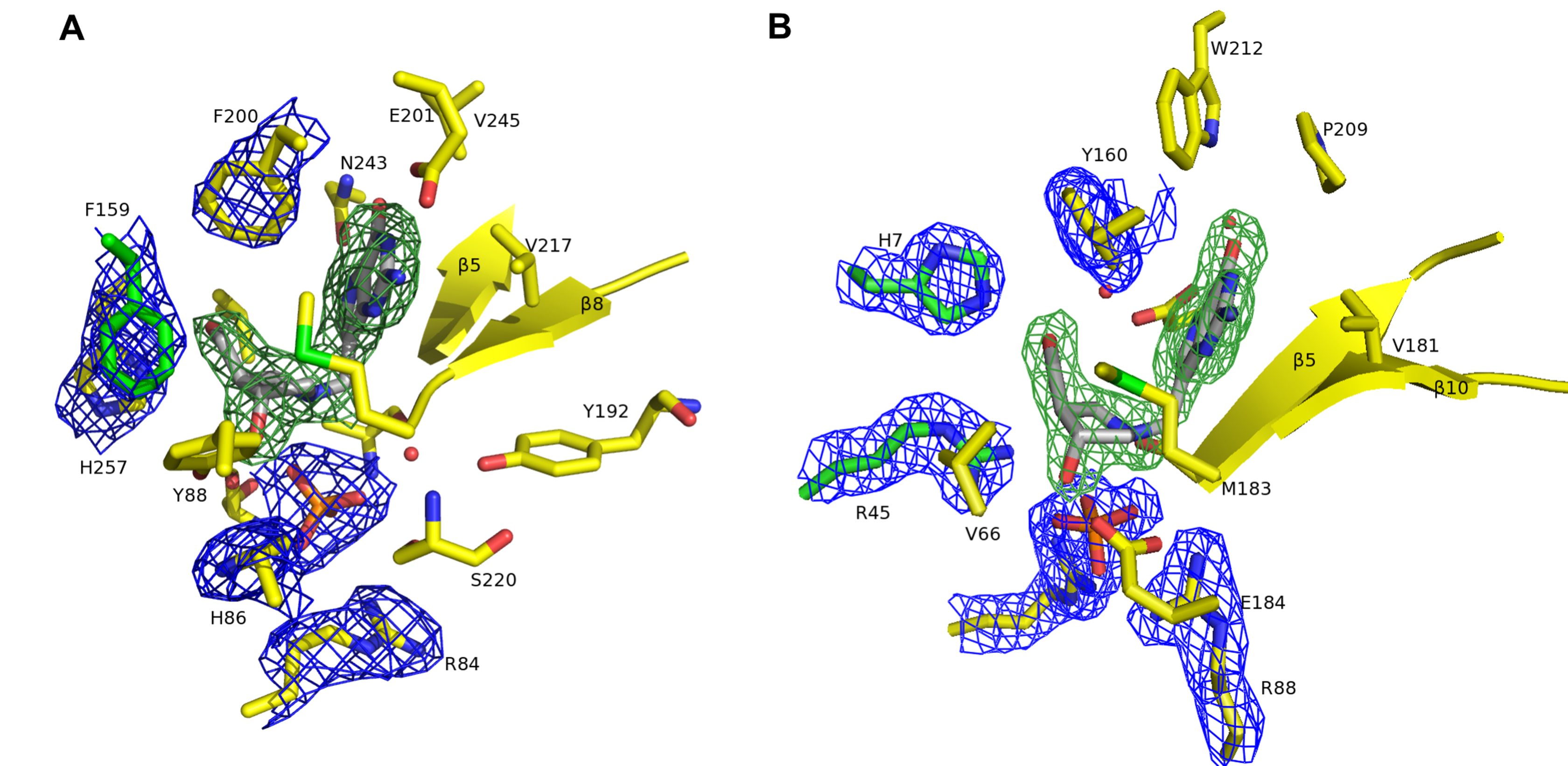

Supplement: Figure S7 — The BCX4945-omitted electron density maps of hPNP and Pf PNP bound to BCX4945. (A) hPNP-BCX4945-PO4 and (B) PfPNP-BCX4945-PO4. BCX4945 (grey), active site residues (yellow), residues from adjacent subunits (green) and phosphate (orange) are indicated. The BCX4945-omitted mFo - DFc electron density map (contour at the 3 σ) is drawn in green. The partial BCX4945-omitted 2mFo - DFc electron density map (contour at the 1 σ) is drawn in blue. (TIF) [file pone.0026916.s007.tif]
