# Supplementary material for: Plasmodium falciparum Parasites Are Killed by a Transition State Analogue of Purine Nucleoside Phosphorylase in a Primate Animal Model
Source: PLoS One. 2011 Nov 11;6(11):e26916. doi: 10.1371/journal.pone.0026916 (PMC3214022; doi:10.1371/journal.pone.0026916)
Supplement: Table S2 — Data collection and refinement statistics. (DOC) [file pone.0026916.s009.doc]

**Table S2.** Data collection and refinement statistics

|  | **hPNP-BCX4945** | ***Pf*PNP-BCX4945** |
| --- | --- | --- |
| **PDB codes** | **3PHB** | **3PHC** |
| **Data collection** |  |  |
| Space group | C2 | P1 |
| Cell dimension |  |  |
| a, b, c (Å) | 269.3, 52.6, 128.1 | 61.2, 77.4, 92.2 |
| α, β, γ (º) | 90.0, 90.3, 90.0 | 67.7, 73.6, 86.0 |
| Resolutions (Å) | 20.00-2.30  (2.38-2.30) | 20.00-2.00  (2.07-2.00) |
| R_sym_ (%) | 9.3 (63.8) | 5.9 (15.2) |
| I / σI | 12.1 (1.6) | 18.6 (7.0) |
| Completeness (%) | 99.3 (94.9) | 94.2 (78.9) |
| Redundancy | 3.9 (3.6) | 3.2 (3.1) |
| **Refinement** |  |  |
| Resolution (Å) | 20.00-2.30 | 20.00-2.00 |
| No. unique reflections | 79666 | 95774 |
| *R*_work_ / *R*_free_ (%) | 23.0/29.5 | 19.8/23.5 |
| **B-factors (Å^2^)** |  |  |
| Protein  (*main chain*)  (*side chain*) | 46.7  48.2 | 28.5  30.8 |
| Water | 32.9 | 29.2 |
| Ligand | 35.8 | 29.2 |
| **No. of Atoms** |  |  |
| Protein | 13462 | 11166 |
| Water | 90 | 312 |
| Ligand | 150 | 150 |
| **R.m.s deviations**  Bond lengths (Å)  Bond angles (º) | 0.013  1.57 | 0.011  1.32 |
| **Ramachandran analysis** |  |  |
| favoured region | 97.2% | 97.5% |
| allowed region | 2.8% | 2.5% |
| Coordinate Error by Luzzati plot (Å) | 0.43 | 0.24 |
| Average Cα r.m.s.d between each subunit | 0.4 Å | 0.1 Å |

Numbers in parentheses are for the highest-resolution shell. One crystal was used for each data set.
