# Supplementary material for: Plasmodium falciparum Parasites Are Killed by a Transition State Analogue of Purine Nucleoside Phosphorylase in a Primate Animal Model
Source: PLoS One. 2011 Nov 11;6(11):e26916. doi: 10.1371/journal.pone.0026916 (PMC3214022; doi:10.1371/journal.pone.0026916)
Supplement: Table S3 — Residues in the active sites of human and PfPNP. (DOC) [file pone.0026916.s010.doc]

**Table S3. Residues in the active sites of human and *Pf*PNP[[1]](#footnote-2)**

|  | **Human PNP** | ***Pf*PNP** |
| --- | --- | --- |
| **Purine** | Ala116  Ala117  Gly118  Phe200  Glu201  Val217  Gly218  Met219  Asn243  Val245 | Ser91  Cys92  Gly93  Tyr160  Val181  Glu182  Met183  Pro209  Asp206  Trp212 |
| **Ribose** | Tyr88  Phe159*****  Phe200  Met219  His257  Val260 | His7*****  Val66  Tyr160  Met183  Glu184  Asp206 |
| **Phosphate** | Ser33  Arg84  His86  Ala116  Tyr192  Ser220 | Gly23  Arg27  Arg45*****  Arg88  Ser91 |

***** The residues are contributed from the adjacent subunit.

1. Mao, C. *et al.* The crystal structure of *Escherichia coli* purine nucleoside phosphorylase: a comparison with the human enzyme reveals a conserved topology. *Structure* **5**, 1373-1383 (1997). [↑](#footnote-ref-2)
