# Supplementary material for: Plasmodium falciparum Parasites Are Killed by a Transition State Analogue of Purine Nucleoside Phosphorylase in a Primate Animal Model
Source: PLoS One. 2011 Nov 11;6(11):e26916. doi: 10.1371/journal.pone.0026916 (PMC3214022; doi:10.1371/journal.pone.0026916)
Supplement: Table S4 — MRM acquisition settings for purines and BCX4945 quantitation. (DOC) [file pone.0026916.s011.doc]

**Table S4. MRM acquisition settings for purines and BCX4945** quantitation

| **Compound** | ***m/z*** | **Cone voltage (V)** | **Collision energy (eV)** |
| --- | --- | --- | --- |
| MTA | 298.2 >136.1 | 24 | 16 |
| MTI | 299.1 >163.0 | 12 | 8 |
| adenosine | 268.1 >136.1 | 24 | 16 |
| inosine | 269.1 >137.1 | 14 | 12 |
| 2′-deoxyguanosine | 268.1 >152.0 | 14 | 12 |
| hypoxanthine | 137.1 >110.0 | 42 | 18 |
| BCX4945 | 280.1 >118.1 | 26 | 14 |
| [2, 8-^2^H]adenosine | 270.1 >138.1 | 24 | 18 |
| [2, 8-^2^H]inosine | 271.1 >139.0 | 74 | 10 |
| [2, 8-^2^H]hypoxanthine | 139.1 >111.8 | 100 | 22 |
| [*methylene*-^2^H_2_]BCX4945 | 282.2 >118.1 | 24 | 14 |
